# Influencer Marketing in Rural Tourism: Enhancing Destination Image and Encouraging Homestay Choices

**DOI:** 10.12688/f1000research.173947.1

**Published:** 2026-01-08

**Authors:** Rohit Chauhan, Alka Shaktan, Ankit Shukla, Kongkiat Khunsathitchai, Nagendra Yadav, Jagadeesh Savanurmath

**Affiliations:** 1 Mittal School of Business, Lovely Professional University, Phagwara, Punjab, 144402, India; 2 Chitkara School of Psychology and Counseling, Chitkara University, Rajpura, Punjab, 140401, India; 3 Faculty of Liberal Arts, Rajamangala University of Technology, Thanyaburi, Thailand; 4 Faculty of Liberal Arts, Rajamangala University of Technology, Thanyaburi, Thailand; 5 Welcomgroup Graduate School of Hotel Administration, Manipal Academy of Higher Education, Manipal, India; 6 Welcomgroup Graduate School of Hotel Administration, Manipal Academy of Higher Education, Manipal, India

**Keywords:** Travel Influencers; Rural Tourism; Homestay Intentions; Destination Image; Theory of Planned Behaviour (TPB); Influencer endorsement; Rural image; Subjective norm

## Abstract

Rural tourism remains underexplored compared to urban and luxury destinations, yet it offers significant potential for sustainable development. Travel influencers, through digital storytelling and endorsements, are increasingly shaping tourist perceptions and reducing uncertainties about homestays. This study examines the impact of influencer endorsements on homestay intentions, utilizing the Theory of Planned Behavior as a guiding framework. Data from 216 urban tourists in Northern and Central India were analyzed with structural equation modeling. Findings reveal that rural image perception is the strongest predictor of homestay intentions, while subjective norms play a weaker but significant role. Influencer endorsements affect behaviour indirectly by enhancing destination image rather than exerting direct persuasion. The study advances the Theory of Planned Behavior by demonstrating the primacy of cognitive evaluations over normative pressures in the context of rural tourism. Practical implications underscore the need for destination marketers and homestay operators to utilize authentic storytelling and image-building strategies to effectively promote rural tourism.

## 1. Introduction

The digital revolution has fundamentally transformed how travellers make decisions, shifting power from traditional brochures to social media influencers who craft compelling narratives about destinations. These modern storytellers wield unprecedented influence through immersive content that shapes perceptions and behaviours ( Dong et al., 2024; Wang et al., 2025). Particularly in rural tourism, influencers serve as vital bridges, using authentic storytelling to showcase cultural richness and counter negative stereotypes ( Y. Li et al., 2023; Qeidari et al., 2024). Their endorsements create social proof that reduces uncertainty about unconventional accommodations, while first-hand experiential insights help overcome perceived risks ( Pramanik, 2023). Yet despite this growing influence, research has largely overlooked how these digital narratives interact with rural image formation and social pressures to drive homestay adoption.

While existing studies have explored influencer marketing’s broad effects ( Dong et al., 2024) and social media’s role in destination branding ( X. Yang et al., 2024), critical gaps remain in understanding rural tourism contexts. Most research focuses on urban or luxury accommodations ( Xu (Rinka) & Pratt, 2018), neglecting how influencer content shapes rural perceptions. Similarly, while subjective norms significantly impact sustainable tourism behaviors ( Liu et al., 2020), their interaction with influencer-driven homestay adoption remains unexamined. This study addresses these gaps through the Theory of Planned Behavior framework, revealing how influencers shape attitudes via rural image building, normalize choices through social validation, and mitigate risks through experiential storytelling ( Girish et al., 2023). By illuminating these psychological mechanisms, we provide crucial insights for leveraging digital influence in rural tourism development.

The primary aim of this study is to examine the role of travel influencers in shaping tourists’ perceptions and behaviours regarding homestays in rural areas. Specifically, this research seeks to: 1) Investigate the extent to which endorsements from travel influencers influence the perceived image of rural areas as tourism destinations. 2) Evaluate how the perceived image of rural areas and subjective norms affect tourists’ intentions to stay in homestays. 3) Determine whether the perceived image of rural areas mediates the effect of travel influencer endorsements on Homestaying intentions. 4) Derive actionable recommendations for leveraging travel influencer endorsements and promoting rural destinations to enhance Homestaying intentions. By addressing these objectives, this study contributes to the growing discourse on digital influence in tourism marketing, rural tourism development, and consumer decision-making models within the TPB framework.

This study is particularly relevant in the context of rural tourism, where promoting non-traditional accommodations like homestays remains a challenge. The research focuses on how travel influencers, through visual storytelling and personal narratives, shape rural image perceptions and create social validation for homestay experiences. Additionally, by applying the Theory of Planned Behaviour, this study situates influencer endorsements within a broader psychological and social framework, examining how attitudes, subjective norms, and perceived rural image collectively influence behavioural intentions toward homestays ( Ajzen, 1991). Furthermore, the study provides practical insights for tourism boards, destination marketers, and hospitality providers on how to optimize influencer collaborations to enhance the appeal of rural destinations. By exploring both the direct and indirect effects of influencer endorsements, the research not only contributes to theoretical advancements in tourism marketing and behavioural psychology but also provides a strategic roadmap for sustainable rural tourism development.

## 2. Literature review

Travel influencer endorsements have emerged as a transformative force in shaping tourists’ perceptions of destinations, particularly in the context of rural tourism. Through digital storytelling and social media engagement, influencers craft narratives that highlight the cultural authenticity, scenic beauty, and immersive experiences of rural areas. For instance, virtual endorsers dressed in culturally specific attire have been shown to significantly enhance tourists’ favourability toward a destination by leveraging symbolic interactionism and processing fluency theory ( Wang et al., 2025). Similarly, ethnic minority endorsers on social media amplify perceptions of authenticity, especially when there is a strong congruence between the influencer and the destination ( Dong et al., 2024). However, the credibility of these endorsements can be fragile; scandals involving influencers can tarnish the cognitive and affective dimensions of a destination’s image, underscoring the delicate balance required in influencer marketing ( X. Yang et al., 2024). These dynamics highlight the strategic role of travel influencers in constructing a compelling rural image, which serves as a foundation for attracting tourists and fostering homestay engagement.

The rural image itself is a multifaceted construct that plays a pivotal role in shaping tourists’ intentions to choose homestays. Rooted in the cognitive-affective-conative (C-A-C) framework, the rural image is shaped by tourists’ perceptions of natural beauty, cultural richness, and the authenticity of rural life ( Pramanik, 2023). Social media and user-generated content, particularly photographs, further reinforce this image by linking cognitive concepts with emotional responses, creating a vivid and relatable portrayal of rural destinations ( K. Zhang et al., 2024). For example, in Iran, visual narratives crafted by foreign tourists have been shown to shape the “tourist gaze,” reinforcing idyllic representations of rural landscapes and fostering a sense of connection ( Qeidari et al., 2024). In China, the evolving roles of rural residents as tourism operators have also influenced the rural image, with machine learning analysis of online reviews revealing shifting expectations around quality and authenticity ( Y. Li et al., 2023). As these perceptions solidify, the rural image becomes a powerful mediator, influencing tourists’ preferences for homestays and underscoring the importance of curated storytelling in rural tourism promotion.

Social influences, encapsulated in the concept of subjective norms, further shape tourists’ decisions to opt for homestays over conventional lodging. Subjective norms, defined as the perceived social pressure to engage in a particular behaviour, have been widely studied through the Theory of Planned Behaviour (TPB). In the context of sustainable coastal tourism, for instance, subjective norms, alongside personal norms and attitudes, have been found to drive responsible tourist behaviour, highlighting the role of social expectations in shaping travel choices ( Liu et al., 2020). Similarly, in green tourism, subjective norms positively influence consumption attitudes, encouraging tourists to make environmentally friendly decisions, such as choosing sustainable accommodations ( Luong, 2023). During the COVID-19 pandemic, subjective norms played a significant role in tourists’ decisions to visit travel bubble destinations, demonstrating their relevance even in health-related decision-making contexts ( Girish et al., 2023). These findings collectively illustrate how subjective norms, whether directly or indirectly, shape tourists’ intentions, often mediated by personal norms or other psychological factors, and reinforce the importance of understanding social influences in tourism management.

Homestaying intentions are deeply rooted in a combination of psychological, social, and experiential factors. Psychological ownership, where tourists develop a sense of control and personal investment in homestays, has been shown to foster long-term loyalty and sustainable behaviours ( Kumar & Chandra, 2024). The concept of “homeyness,” characterized by familiarity, authenticity, and security, further enhances tourists’ satisfaction and encourages pro-environmental behaviors, making homestays an attractive option for those seeking meaningful travel experiences ( Baratta et al., 2024). Technological advancements, such as optimized genetic algorithms for personalized recommendations, have also been shown to increase homestay bookings, highlighting the growing role of technology in shaping tourists’ choices ( Suanpang et al., 2024). Additionally, the COVID-19 pandemic amplified tourists’ preferences for local homestay options, driven by risk aversion and a desire for community attachment, further underscoring the psychological and social underpinnings of homestay motivations ( Pichierri, 2023). These insights collectively illustrate how travel influencer endorsements, rural image, and subjective norms interact to foster strong Homestaying intentions, ensuring a seamless transition from digital influence to real-world travel choices.

### 2.1 Hypothesis formulation

The increasing role of travel influencers in shaping tourism choices has positioned them as key opinion leaders in digital spaces. Their endorsements significantly influence potential travellers’ decision-making processes by providing authentic and visually engaging narratives that highlight cultural experiences, local hospitality, and immersive stays. Social media platforms amplify this influence, as influencer-generated content enhances tourists’ confidence in choosing homestays by offering detailed insights into accommodations, local attractions, and unique experiences ( Wang et al., 2025). Furthermore, research suggests that influencer credibility—comprising expertise, trustworthiness, and attractiveness—directly affects tourists’ intentions to visit endorsed locations ( Dong et al., 2024). By promoting rural homestays through personal experiences and storytelling, influencers reduce perceived risks associated with non-traditional accommodations and encourage their followers to opt for these alternative lodging options. Given this persuasive effect, it is expected that influencer endorsements significantly enhance tourists’ willingness to stay in homestays.

H1: Travel influencer endorsements positively impact tourists’ Homestaying intentions.

While influencer endorsements play a direct role in influencing accommodation choices, their effectiveness is often mediated by the perceived image of the rural destination. Tourists form cognitive perceptions of rural environments based on the portrayal of scenic beauty, cultural richness, and hospitality in influencer-generated content. These perceptions shape affective responses, which in turn drive behavioral intentions, as explained by the cognitive-affective-conative (C-A-C) framework ( Pramanik, 2023). Additionally, the reinforcement of rural imagery through social media and user-generated content strengthens tourists’ emotional connections with homestays ( K. Zhang et al., 2024). Research also highlights that an appealing rural image fosters a sense of escapism and authenticity, making tourists more inclined toward choosing homestays over conventional lodging options. Therefore, a strong and positive rural image is expected to encourage homestay adoption.

H2: Perceived rural image positively affects tourists’ Homestaying intentions.

Beyond destination perceptions, social influences play a significant role in shaping travel behavior. Subjective norms, defined as the perceived social pressure to engage in a particular behavior, are critical in influencing accommodation choices. Tourists often consider homestays when they perceive endorsement from peers, family, or influencers ( Ajzen, 1991). Previous research indicates that subjective norms impact sustainable tourism behaviours, such as choosing eco-friendly accommodations and participating in community-based tourism ( Luong, 2023). The growing popularity of rural tourism, driven by social media discourse, has further normalized homestay experiences as a preferred lodging option. When travellers feel that homestay accommodations align with socially accepted and recommended travel trends, they are more likely to choose them over hotels.

H3: Subjective norms positively influence tourists’ Homestaying intentions.

The influence of travel influencers extends beyond direct persuasion and plays a crucial role in shaping subjective norms. When influencers endorse homestays, they contribute to establishing new social norms that validate alternative lodging options. Their narratives and personal experiences create a perception that homestays are socially accepted, enriching, and desirable accommodations ( Liu et al., 2020). This is particularly significant in emerging travel trends, where influencer-driven content shapes aspirational travel choices. By presenting homestays as immersive and culturally enriching experiences, influencers reinforce positive social validation, which in turn influences tourists’ willingness to consider homestays. This suggests that subjective norms mediate the relationship between influencer endorsements and homestay intentions.

H4: Subjective norms mediate the relationship between travel influencer endorsements and Homestaying intentions.

In addition to subjective norms, influencer endorsements also contribute to shaping the perceived rural image, which further influences homestay choices. The portrayal of rural destinations in influencer content—through engaging narratives, high-quality visuals, and cultural storytelling—plays a crucial role in forming tourists’ perceptions. Studies indicate that destination image significantly affects travel decisions, as it shapes expectations and perceived value ( T. Yang et al., 2023). Furthermore, research suggests that destination image acts as a mediator, strengthening the link between influencer promotions and tourists’ behavioural outcomes ( Qeidari et al., 2024). Since perceived rural image is a key determinant of homestay preferences, it is expected to mediate the relationship between influencer endorsements and Homestaying intentions.

H5: Perceived rural image mediates the relationship between travel influencer endorsements and Homestaying intentions.

**Figure 1.**
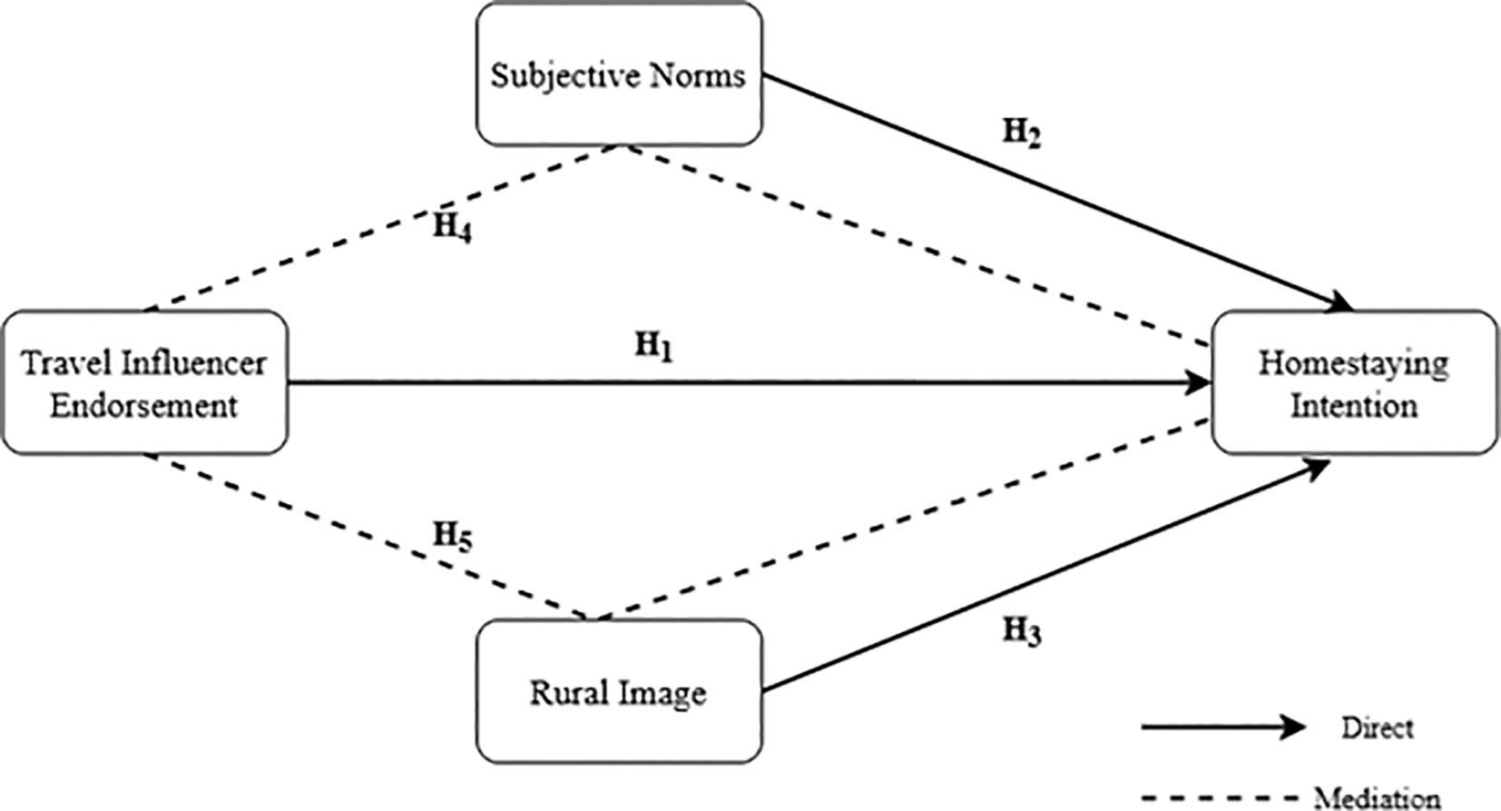
The conceptual framework (Source: Authors).

Figure 1 presents the conceptual framework showing relationships between travel influencer endorsement, rural image, subjective norms, and Homestaying intentions.

## 3. Research methodology

This study was conducted in Northern and Central India, regions known for their rich cultural heritage, diverse rural attractions, and emerging tourism potential. Specifically, data collection took place in urban centers within six major cities: Patna, Delhi, Jalandhar, Chandigarh, Ambala, and Jammu. These cities were strategically selected due to their significant urban populations, which serve as key sources of tourists for rural homestays. The rationale behind choosing these locations is grounded in previous research that emphasizes the importance of cultural and rural attractions in shaping tourists’ preferences for homestays ( Hung et al., 2021). Furthermore, the psychological attachment tourists develop towards homestays in these regions contributes to sustainable tourism behaviours ( Kumar & Chandra, 2024). The impact of travel influencer endorsements, particularly on digital platforms, also plays a crucial role in shaping urban tourists’ perceptions of rural tourism destinations ( P. Li & Sun, 2024). Given the growing importance of social media in tourism decision-making ( Pop et al., 2022), these cities provided an ideal sample for analyzing how influencer endorsements, subjective norms, and rural image contribute to homestay intentions among urban tourists.

A simple random sampling method was employed to ensure an unbiased representation of urban tourists who had undertaken a tour in the past year. In each of the six selected cities, data collection was carried out with the assistance of trained enumerators, who were supervised by one of the co-authors. Before participating, all respondents were fully informed about the study’s aims and objectives. This study did not involve any sensitive personal data collection, clinical procedures, or interventions. This research was designed and conducted in accordance with the ethical principles of the Declaration of Helsinki and the ICMR National Ethical Guidelines for Biomedical and Health Research Involving Human Participants (2017) ( ICMR, 2017). No personally identifiable information was collected at any stage, ensuring complete anonymity of respondents. Prior to data collection, informed consent was obtained from all participants. A written consent checkbox was provided at the start of the online questionnaire, clearly stating the purpose of the study, voluntary nature of participation, confidentiality of responses, and exclusive use of data for academic research. Participants were explicitly informed that they could discontinue participation at any point without any justification, and no data would be retained if they chose to withdraw. These measures ensured adherence to national ethical standards for research involving human respondents.

To enhance the reliability and accuracy of data collection, an online questionnaire was used, which the enumerators filled out during face-to-face interactions with respondents. This method enabled real-time clarification of any doubts and resulted in a higher response rate. The questionnaire targeted individuals who had traveled within the past year, ensuring that their experiences and perceptions of rural homestays were recent and relevant. Given the role of digital media in shaping tourism decisions, the study considered responses from individuals actively engaged with social media travel influencers, thus capturing the influence of digital endorsements on homestay intentions ( P. Li & Sun, 2024). The sample size for this research was found adequate for 16 items and 216 responses. The response to the item ratio is greater than 10, satisfying the threshold suggested by Hair et al. (2019).

The survey instrument employed in this study was adapted from validated scales used in prior research. A five-point Likert scale was used to measure key constructs such as travel influencer endorsement ( Shoukat et al., 2023), subjective norms ( Bianchi & Mortimer, 2015), rural image ( Beerli & Martín, 2004), and homestay intentions ( Kock et al., 2019). The content validity of the instrument was verified by two university professors with expertise in tourism and consumer behavior, as well as three professional travel planners familiar with homestay tourism. Additionally, a pilot test was conducted on the first 45 respondents to assess the instrument’s reliability. The results indicated strong internal consistency, with Cronbach’s alpha values exceeding the acceptable threshold of 0.7, confirming the reliability of the measures. This rigorous approach to survey design and validation ensures that the findings accurately reflect the influence of travel influencer endorsements, subjective norms, and rural image on tourists’ homestay intentions.

## 4. Results

### 4.1 Demographic profile

The demographic profile of the respondents provides a clear overview of the sample population for the study on the impact of travel influencer endorsement, subjective norm, and rural image on homestaying intention. The sample consists of 216 respondents, with a nearly equal gender distribution: 52.8% females (114 respondents) and 47.2% males (102 respondents). In terms of age, the majority of respondents (77.8%, 168 respondents) fall within the 18-35 age group, indicating a strong representation of younger individuals. The 36-55 age group constitutes 17.6% (38 respondents), while those above 55 years account for 3.7% (8 respondents). Regarding education, most respondents hold a Graduation/Bachelor’s degree (55.1%, 119 respondents), followed by Post-graduation/Master’s degree holders (36.6%, 79 respondents). A smaller percentage have a Doctorate (3.2%, 7 respondents) or education up to the school level (5.1%, 11 respondents). This demographic profile suggests that the sample is predominantly young, well-educated, and balanced in terms of gender, which may influence their perceptions and decision-making regarding Homestaying intentions.

### 4.2 Measurement model results

The measurement model was rigorously evaluated to ensure the reliability, validity, and overall fit of the constructs used in the study. The results of measurement model are presented in Table 1. This evaluation involved assessing convergent validity, discriminant validity, and model fit indices, each of which serves a specific purpose in confirming the robustness of the measurement model. The results demonstrate that the constructs are reliable, distinct, and well-suited for testing the hypothesized relationships. Convergent validity was assessed using Standardized Factor Loadings (SFL), Composite Reliability (CR), and Average Variance Extracted (AVE). These metrics were calculated to ensure that the items measuring each construct are strongly correlated and that the constructs themselves are reliable and valid. The SFL values for all items exceeded the recommended threshold of 0.7 ( Hair et al., 2019), indicating that the items are strong indicators of their respective constructs. The CR values, which measure internal consistency, ranged between 0.877 and 0.933, surpassing the threshold of 0.7 ( Fornell & Larcker, 1981). This confirms that the constructs are internally consistent and reliable. The AVE values, which quantify the amount of variance captured by a construct relative to measurement error, ranged from 0.641 to 0.751, exceeding the minimum threshold of 0.5 ( Fornell & Larcker, 1981). This indicates that the constructs explain a significant portion of the variance in their items, supporting convergent validity.

**Table 1.** Measurement model results (Source: Authors).

Constructs and items	SFL	CR	AVE
**Travel influencer endorsement** ( Shoukat et al., 2023)		0.888	0.665
I often watch influencer videos to be aware of rural homestays' feedback from others.	0.872		
To make sure I visit the right rural homestay, I often view social media influencers' videos.	0.825		
I frequently gather information from social media videos to help me choose the right rural homestay	0.777		
When I visit a rural space, influencers' endorsement makes me confident in my choice.	0.783		
**Subjective Norms** ( Bianchi & Mortimer, 2015)		0.933	0.736
*I should stay in a homestay because*			
My friends think that	0.869		
People who are important to me think that	0.877		
People who influence my consumer behaviour think that	0.872		
Society thinks that that	0.877		
My family thinks that	0.792		
**Rural Image** ( Beerli & Martín, 2004)		0.877	0.641
*Rural areas of my state provide following as a tourism destination for tourists*			
rich culture	0.758		
rich flora and fauna	0.897		
Interesting cultural activities	0.776		
varied gastronomy	0.764		
**Homestaying intention** ( Kock et al., 2019)		0.9	0.751
I intend to stay at a homestay on my next holiday to a rural destination.	0.859		
The next time I go on vacation to a rural destination, I will stay at a homestay.	0.876		
It is very likely that I would choose to stay at a homestay when I travel to a rural destination	0.865		

**SFL**: Standardized Factor Loading; **AVE**: Average Variance Extracted; **CR**: Composite Reliability.

**Model fit**: CMIN/DF: 1.856; GFI: 0.904; IFI: 0.965; TLI: 0.957; CFI: 0.965; RMR: 0.052; RMSEA: 0.063; SRMR: 0.0462.

The overall fit of the measurement model was evaluated using a range of fit indices, which are categorized into goodness-of-fit and badness-of-fit measures. These indices were calculated to determine how well the hypothesized model aligns with the observed data. Goodness-of-fit measures include the Goodness-of-Fit Index (GFI), Incremental Fit Index (IFI), Tucker-Lewis Index (TLI), and Comparative Fit Index (CFI). These indices indicate the proportion of variance explained by the model, with values above 0.90 considered acceptable ( Hu & Bentler, 1999). In this study, the GFI (0.904), IFI (0.965), TLI (0.957), and CFI (0.965) all exceeded the threshold, confirming a good fit. Badness-of-fit measures include the Root Mean Square Residual (RMR), Standardized Root Mean Square Residual (SRMR), and Root Mean Square Error of Approximation (RMSEA). These indices quantify the discrepancy between the observed and predicted covariance matrices, with lower values indicating a better fit. The RMR (0.052), SRMR (0.0462), and RMSEA (0.063) were all below their respective thresholds of 0.08 ( Hu & Bentler, 1999), further supporting the model’s fit. Additionally, the CMIN/DF (Chi-square to degrees of freedom ratio) was calculated to assess the model’s parsimony. A value of 1.856 was obtained, which is well below the threshold of 3 ( Kline & Rex B, 2015), indicating an excellent balance between model complexity and fit.

Discriminant validity was assessed using the Fornell-Larcker Criterion ( Fornell & Larcker, 1981), which ensures that each construct is distinct from the others and are presented in Table 2. This criterion requires that the square root of the AVE for each construct should be greater than the correlations between that construct and all other constructs. In this study, the square roots of the AVE values were higher than the off-diagonal correlations, confirming discriminant validity. For example, the square root of AVE for Travel Influencer Endorsement (0.815) was greater than its correlations with other constructs (ranging from 0.380 to 0.553). Similar results were observed for all other constructs, ensuring that each construct is unique and captures a distinct aspect of the research model.

**Table 2.** Discriminant validity using Fornell-Larcker Criteria (Source: Authors).

	Travel Influencer Endorsement	Subjective Norms	Rural Image	Homestaying Intention
** Travel Influencer Endorsement**	**0.815**			
**Subjective Norms**	0.381***	**0.858**		
**Rural Image**	0.380***	0.255**	**0.801**	
**Homestaying Intention**	0.553***	0.368***	0.580***	**0.867**

** and *** denote statistical significance at p<0.01 and p<0.001 levels, indicating stronger confidence in relationships.

### 4.3 Structural model results

The direct effect of Travel Influencer Endorsement on Rural Image was significant (Estimate = 0.32, S.E. = 0.06, C.R. = 4.95, p < 0.001), indicating that endorsements from travel influencers positively enhance the perceived image of rural areas as tourism destinations. Similarly, the direct effect of Rural Image on Homestaying Intention was also significant (Estimate = 0.47, S.E. = 0.08, C.R. = 5.82, p < 0.001), demonstrating that a positive perception of rural areas significantly increases the intention to stay in homestays. Additionally, the direct effect of Subjective Norms on Homestaying Intention was significant but relatively weaker (Estimate = 0.10, S.E. = 0.05, C.R. = 2.11, p = 0.04), suggesting that social influence from friends, family, and society plays a modest but statistically significant role in shaping Homestaying intentions.

The mediation analysis, conducted using bootstrapping with 5,000 samples at a 95% confidence interval (CI), revealed significant direct and indirect effects, providing a comprehensive understanding of the relationships between the independent variables, mediators, and the dependent variable. The mediation analysis further revealed that Rural Image significantly mediates the relationship between Travel Influencer Endorsement and Homestaying Intention (Estimate = 0.15, S.E. = 0.05, 95% CI = [0.07, 0.28], C.R. = 2.11, p < 0.001), indicating that travel influencers’ endorsements enhance the perceived image of rural areas, which in turn increases the likelihood of choosing homestays. On the other hand, the mediating role of Subjective Norms was also significant but smaller in magnitude (Estimate = 0.05, S.E. = 0.03, 95% CI = [0.01, 0.11], C.R. = 2.98, p = 0.03), suggesting that while travel influencers’ endorsements influence social norms, their impact on Homestaying intentions is relatively limited compared to the effect mediated by Rural Image. The result of direct and mediating relationships is presented in Table 3.

**Table 3.** Results of path analysis (Source: Authors).

	Independent variable	Mediator	Dependent variable	Estimate	S.E.	Bootstrap 95% CI		C.R.	P	Supported
						Lower	Upper			
H1	Travel Influencer Endorsement		Homestaying Intention	0.32	0.06			4.95	***	Yes
H2	Subjective Norms		Homestaying Intention	0.10	0.05			2.11	0.04 *	Yes
H3	Rural Image		Homestaying Intention	0.47	0.08			5.82	***	Yes
H4	Travel Influencer Endorsement	Subjective Norms	Homestaying Intention	0.05	0.03	0.01	0.11	2.98	0.03 *	Yes
H5	Travel Influencer Endorsement	Rural Image	Homestaying Intention	0.15	0.05	0.07	0.28	2.11	***	Yes

*** p<.001;

* p<.05; SE: Standard Error; CI: Confidence Intervals; CR: Critical ratio; p: Significance.

Overall, the findings highlight the dual role of Travel Influencer Endorsement in directly and indirectly influencing Homestaying Intention, with Rural Image emerging as a stronger mediator compared to Subjective Norms. These results underscore the importance of leveraging travel influencers to promote rural destinations and enhance their appeal, as well as the need to consider social influences in shaping travel intentions. The study provides valuable insights for tourism marketers and policymakers, emphasizing the strategic use of influencer endorsements and the promotion of rural areas’ unique cultural and environmental attributes to boost Homestaying intentions.

## 5. Conclusion and discussion

The findings of this study strongly align with the Theory of Planned Behaviour (TPB), demonstrating that travel influencer endorsements influence homestay intentions through cognitive and normative mechanisms. The study confirms that attitudes, shaped by rural image perception, play a dominant role in behavioural intentions, with rural image emerging as the strongest predictor. This aligns with previous research suggesting that destination image is a crucial attitudinal driver of travel decisions ( Pramanik, 2023). While subjective norms also influence homestay intentions, their weaker impact suggests that social influence is secondary to personal evaluations in rural tourism contexts. Instead, these findings suggest that rural tourism decisions are more self-driven, where personal attitudes and perceptions outweigh societal expectations. Thus, enhancing rural image through strategic influencer marketing is more effective than merely leveraging social influence to drive homestay adoption.

A key distinction from previous studies is the mediating role of rural image in influencer-driven travel decisions. This study highlights that influencers play a more indirect but equally crucial role in shaping travellers’ mental perceptions of rural destinations. Unlike urban tourism, where influencers often act as direct persuaders, in rural tourism, their role is more about storytelling and image-building. For instance, virtual endorsers dressed in culturally specific attire have been shown to significantly enhance tourists’ favourability toward a destination by leveraging symbolic interactionism and processing fluency theory ( Wang et al., 2025). Similarly, ethnic minority endorsers on social media amplify perceptions of authenticity, especially when there is a strong congruence between the influencer and the destination ( Dong et al., 2024). These dynamics highlight the strategic role of travel influencers in constructing a compelling rural image, which serves as a foundation for attracting tourists and fostering homestay engagement.

Moreover, while the Theory of Planned Behaviour (TPB) traditionally emphasizes subjective norms as a key social pressure influencing decisions, the weaker effect of subjective norms here suggests that tourists in rural settings are not as dependent on peer influence as those choosing mainstream destinations. This implies that in rural tourism, destination branding and authentic narratives hold greater sway over decisions than simple social validation. For example, in sustainable coastal tourism, subjective norms, along with personal norms and attitudes, have been found to drive responsible tourist behaviour, highlighting the role of social expectations in shaping travel choices ( Liu et al., 2020). Similarly, in green tourism, subjective norms positively influence consumption attitudes, encouraging tourists to make environmentally friendly decisions, such as choosing sustainable accommodations ( Luong, 2023). However, the limited role of subjective norms in this study suggests that rural tourism decisions are more influenced by the cognitive and affective dimensions of the rural image, as shaped by influencer endorsements and experiential storytelling.

The results also refine the understanding of TPB’s perceived behavioural control (PBC) component, which was not explicitly tested but is conceptually relevant. While rural tourism is often associated with logistical constraints, accessibility concerns, and unfamiliarity, the fact that rural image perception was a stronger determinant than subjective norms suggests that a positive cognitive evaluation of rural destinations can mitigate perceived barriers. If potential tourists develop a compelling, immersive, and positive mental image of a rural area, they are more likely to commit to visiting, regardless of accessibility concerns. This further reinforces the idea that influencers must go beyond traditional promotional tactics to create deep, emotionally engaging narratives that reshape perceptions of rural destinations. For instance, the cognitive-affective-conative (C-A-C) framework explains how cognitive perceptions of rural environments, such as natural beauty and cultural richness, influence affective responses, which in turn drive behavioural intentions ( Pramanik, 2023). The integration of social media and user-generated content, particularly photographs, reinforces this rural image by linking cognitive concepts with emotional responses, creating a vivid and relatable portrayal of rural destinations ( J. Zhang, 2024).

The overall findings contribute to the evolving discourse on influencer marketing, rural tourism behaviour, and TPB applications beyond urban settings. They highlight that while influencer endorsements create initial awareness, their real impact lies in fostering strong cognitive associations with rural destinations. This challenges previous assumptions that social influence and trust in influencers alone are enough to drive behavioural change. Instead, this study demonstrates that perceived rural image is the most critical factor, requiring destination marketers and influencers to focus on building long-term, experience-driven content strategies. For example, in China, the evolving roles of rural residents as tourism operators have influenced the rural image, with machine learning analysis of online reviews revealing shifting expectations around quality and authenticity ( Y. Li et al., 2023). These insights are particularly valuable for tourism boards, marketers, and policymakers looking to leverage digital media for sustainable rural tourism promotion. By understanding and leveraging these dynamics, destination managers can effectively promote rural tourism and enhance the appeal of homestays, ensuring both economic viability and cultural enrichment.

### 5.1 Theoretical contributions

This study makes key theoretical contributions to the application of the Theory of Planned Behavior (TPB) in rural tourism and influencer marketing contexts. First, it extends TPB by demonstrating that travel influencer endorsements influence behavioral intentions not only through attitudes but also through perceived destination image and subjective norms. Unlike traditional TPB models that primarily focus on direct behavioral antecedents ( Ajzen, 1991), this study illustrates the importance of mediated relationships, where cognitive perceptions of rural destinations significantly influence decision-making. Second, it expands influencer marketing literature by validating the dual role of influencers in both shaping attitudes and reinforcing social norms. Prior research has established that influencer credibility enhances product adoption ( Lou & Yuan, 2019); however, this study broadens this understanding by situating endorsements within a structured behavioral framework. Third, the study provides empirical support for the argument that rural image serves as a stronger determinant of homestay intention than social influence, challenging traditional assumptions about peer-driven decision-making in tourism. By integrating these insights, this research enriches the theoretical discourse on social media influence, rural tourism adoption, and planned behavior theory within the digital landscape.

### 5.2 Practical implications

The findings offer valuable insights for tourism boards, digital marketers, and homestay operators seeking to leverage influencer marketing effectively. First, tourism marketers must recognize that rural image enhancement is critical for influencing homestay adoption. While influencer endorsements help generate interest, destination managers should invest in improving the physical, cultural, and experiential attributes of rural areas to strengthen positive image perceptions. This may include developing immersive tourism experiences, promoting local storytelling, and showcasing heritage tourism initiatives to align with influencer content.

Second, influencer marketing strategies should focus on experiential authenticity rather than generic promotional content. Given that influencers shape perceptions through storytelling, collaborations with local hosts, community-driven content, and firsthand experiences should be prioritized. User-generated content featuring actual homestay guests can further reinforce credibility and authenticity, aligning with the psychological mechanisms of social proof and cognitive consistency. Additionally, since subjective norms have a weaker but still significant effect, targeted marketing campaigns should incorporate community endorsements and peer-based recommendations, such as testimonials from previous homestay guests, user reviews, and interactive social media engagement.

Third, destination managers should explore interactive digital strategies to capitalize on influencer-generated interest. This includes developing virtual rural tourism experiences, influencer-hosted live sessions showcasing rural accommodations, and engaging storytelling through short-form videos. The integration of augmented reality (AR) and virtual reality (VR) experiences could further enhance the perceived appeal of rural homestays by allowing potential tourists to explore destinations before committing to travel. Moreover, influencer partnerships should focus on long-term engagements rather than one-off promotions, ensuring sustained visibility and trust-building.

Finally, policymakers and tourism development authorities should implement training programs for rural homestay providers on digital marketing strategies and influencer collaboration. Many rural tourism entrepreneurs may lack the expertise to leverage digital platforms effectively. By fostering digital literacy and influencer engagement strategies, rural communities can become more self-reliant in promoting their tourism offerings. Additionally, incentivizing influencers through sponsorships, collaborations, and subsidized familiarization trips can increase the representation of rural destinations in mainstream digital narratives, ultimately enhancing the visibility and attractiveness of rural homestays.

### 5.3 Limitations and future scope

This study has certain limitations that open avenues for future research. First, it primarily focuses on a single rural tourism context, limiting the generalizability of findings across diverse rural destinations. Future research should examine regional variations in influencer effectiveness, particularly in areas with different cultural and infrastructural characteristics. Second, this study does not differentiate between different types of influencers (micro vs. macro), which may impact perceived credibility and engagement. Future studies should investigate how influencer size, expertise, and engagement levels influence rural tourism adoption. Finally, this research does not assess the long-term effects of influencer marketing, leaving room for longitudinal studies to explore how sustained influencer engagement affects rural tourism development over time.

## Data Availability

The dataset includes all values used for the statistical analyses, tables, and figures, and comprises responses from 217 participants who took part in the study. The dataset and questionnaire is openly available at https://doi.org/10.6084/m9.figshare.30742610.v2 ( Chauhan, 2025). Data are available under the terms of the Creative Commons Attribution 4.0 International license (CC-BY 4.0).
