# Supplementary material for: Influencer Marketing in Rural Tourism: Enhancing Destination Image and Encouraging Homestay Choices
Source: F1000Res. 2026 Jan 8;15:32. [Version 1] doi: 10.12688/f1000research.173947.1 (PMC12811726; doi:10.12688/f1000research.173947.1)
Supplement: Peer review history

# Reviewer report: Reviewer response for version 1

**DOI:** 10.5256/f1000research.191808.r448987

**Published:** 2026-01-21

**Authors:** Dilbag Singh

**Affiliations:** 1 Starex University, Gurugram, Haryana, India

The article *“Influencer Marketing in Rural Tourism: Enhancing Destination Image and Encouraging Homestay Choices”*is clearly written, well-structured, and cites recent literature from 2023–2025, making it academically current. The study design is appropriate, applying the **Theory of Planned Behavior** and using **structural equation modeling (SEM)** with a sufficient sample size of 216 respondents. Ethical standards are followed, and measurement reliability is confirmed through Cronbach’s alpha and validity checks.

 Methodological details are provided, though replication would benefit from fuller reporting of SEM outputs and mediation effects. Statistical analysis is sound, with appropriate interpretation highlighting that **rural image is the strongest predictor of homestay intentions**, while influencer endorsements work indirectly by enhancing destination image. Subjective norms play a weaker but still significant role.

 The article *“Influencer Marketing in Rural Tourism: Enhancing Destination Image and Encouraging Homestay Choices”*is clearly written, well-structured, and cites recent literature from 2023–2025, making it academically current. The study design is appropriate, applying the **Theory of Planned Behavior** and using **structural equation modeling (SEM)** with a sufficient sample size of 216 respondents. Ethical standards are followed, and measurement reliability is confirmed through Cronbach’s alpha and validity checks.

 Methodological details are provided, though replication would benefit from fuller reporting of SEM outputs and mediation effects. Statistical analysis is sound, with appropriate interpretation highlighting that **rural image is the strongest predictor of homestay intentions**, while influencer endorsements work indirectly by enhancing destination image. Subjective norms play a strong and  significant role.

Is the work clearly and accurately presented and does it cite the current literature?

Yes

If applicable, is the statistical analysis and its interpretation appropriate?

Yes

Are all the source data underlying the results available to ensure full reproducibility?

Yes

Is the study design appropriate and is the work technically sound?

Yes

Are the conclusions drawn adequately supported by the results?

Yes

Are sufficient details of methods and analysis provided to allow replication by others?

Yes

Reviewer Expertise:

I am a hospitality educator serving the nation in the field of hotel management from last 14 years. My area of research are Employee Turnover, Sustainable Practices, Event Management and other fields of hospitality and tourism.

I confirm that I have read this submission and believe that I have an appropriate level of expertise to confirm that it is of an acceptable scientific standard.

# Reviewer report: Reviewer response for version 1

**DOI:** 10.5256/f1000research.191808.r448988

**Published:** 2026-01-21

**Authors:** Abhishek Ghai

**Affiliations:** 1 Maharishi Markandeshwar (Deemed to be University), Mullana-Ambala, Haryana, India

The addresses a timely and underexplored area within tourism marketing by integrating influencer marketing with rural tourism through the Theory of Planned Behavior (TPB). The study is conceptually sound, empirically grounded, and well-positioned to contribute to both tourism and digital marketing literature. The writing is largely clear, and the arguments are logically developed. Overall, the manuscript demonstrates good scholarly rigor and practical relevance. The manuscript has strong potential for publication and it can make a significant contribution to the literature on influencer marketing, rural tourism, and behavioral theory applications.

Is the work clearly and accurately presented and does it cite the current literature?

Yes

If applicable, is the statistical analysis and its interpretation appropriate?

Yes

Are all the source data underlying the results available to ensure full reproducibility?

Yes

Is the study design appropriate and is the work technically sound?

Yes

Are the conclusions drawn adequately supported by the results?

Yes

Are sufficient details of methods and analysis provided to allow replication by others?

Yes

Reviewer Expertise:

Food and Beverage management, event management, tour and travel agency

I confirm that I have read this submission and believe that I have an appropriate level of expertise to confirm that it is of an acceptable scientific standard.

# Reviewer report: Reviewer response for version 1

**DOI:** 10.5256/f1000research.191808.r448983

**Published:** 2026-01-16

**Authors:** Gaurav Bathla

**Affiliations:** 1 CT University Ludhiana, Ludhiana, Punjab, India

The paper addresses a timely and relevant topic by examining the role of influencer marketing in shaping destination image and encouraging homestay choices in rural tourism. The conceptual framework is clearly presented and effectively connects influencer credibility with destination image formation and tourists’ accommodation decisions. The study contributes meaningfully to rural tourism literature by highlighting how digital influencer strategies can support rural destinations and local homestay operators. The methodology is generally appropriate for the stated objectives; however, greater clarity regarding sample selection and analytical procedures would strengthen the rigor of the study. Overall, the findings offer valuable theoretical insights and practical implications for policymakers and rural tourism stakeholders, and with minor refinements, the manuscript demonstrates strong potential for publication.

Is the work clearly and accurately presented and does it cite the current literature?

Yes

If applicable, is the statistical analysis and its interpretation appropriate?

Yes

Are all the source data underlying the results available to ensure full reproducibility?

Yes

Is the study design appropriate and is the work technically sound?

Yes

Are the conclusions drawn adequately supported by the results?

Yes

Are sufficient details of methods and analysis provided to allow replication by others?

Yes

Reviewer Expertise:

Hospitality Management , Hospitality Marketing , Tourism Management

I confirm that I have read this submission and believe that I have an appropriate level of expertise to confirm that it is of an acceptable scientific standard.

# Reviewer report: Reviewer response for version 1

**DOI:** 10.5256/f1000research.191808.r448986

**Published:** 2026-01-16

**Authors:** Pramendra Singh

**Affiliations:** 1 Marwadi University, Rajkot, Gujarat, India

This article presents a thoughtful and well-executed study on the growing role of travel influencers in shaping tourists’ preferences for rural homestays. By grounding the research in the Theory of Planned Behavior and supporting it with solid empirical evidence, the authors clearly show that influencers are most effective when they help build a positive and appealing image of rural destinations rather than simply persuading audiences directly. The analysis is carefully carried out, the arguments are logically developed, and the findings are explained in a clear and relatable manner. Importantly, the study offers useful insights for both researchers and practitioners by showing how authentic storytelling and destination image can encourage interest in rural tourism. The article makes a meaningful contribution to the field and is well suited for publication.

Is the work clearly and accurately presented and does it cite the current literature?

Yes

If applicable, is the statistical analysis and its interpretation appropriate?

Yes

Are all the source data underlying the results available to ensure full reproducibility?

Yes

Is the study design appropriate and is the work technically sound?

Yes

Are the conclusions drawn adequately supported by the results?

Yes

Are sufficient details of methods and analysis provided to allow replication by others?

Yes

Reviewer Expertise:

Sustainable tourism, Heritage tourism, Tourism education, Tourist behaviour

I confirm that I have read this submission and believe that I have an appropriate level of expertise to confirm that it is of an acceptable scientific standard.

# Reviewer report: Reviewer response for version 1

**DOI:** 10.5256/f1000research.191808.r448985

**Published:** 2026-01-16

**Authors:** Ankush Ambardar

**Affiliations:** 1 Central University of Haryana, Mohindergarh, Haryana, India

The study examines how travel influencer endorsements shape tourists’ homestaying intentions in rural areas, using the Theory of Planned Behavior (TPB) as the guiding framework. It addresses a critical gap in tourism literature by focusing on rural homestays, a context often overlooked in favor of urban or luxury accommodations. The research proposes that travel influencers affect homestaying intentions directly and indirectly through two key psychological mechanisms: perceived rural image and subjective norms. Influencer-generated storytelling and visual content enhance the cognitive and affective image of rural destinations, while also normalizing homestays through social validation. Data were collected from 216 urban tourists in Northern and Central India using a structured questionnaire adapted from validated scales. Structural Equation Modeling (SEM) confirmed the reliability and validity of all constructs. The results show that:

- Travel influencer endorsements significantly enhance rural image.
- Rural image strongly predicts homestaying intention.
- Subjective norms also positively influence homestaying intention, though to a lesser extent.
- Rural image is a stronger mediator than subjective norms between influencer endorsement and homestaying intention.

Overall, the study contributes theoretically by extending TPB into digital influencer-driven rural tourism and practically by offering insights for destination marketers and policymakers seeking to promote sustainable rural tourism through influencer collaborations.

Is the work clearly and accurately presented and does it cite the current literature?

Yes

If applicable, is the statistical analysis and its interpretation appropriate?

Yes

Are all the source data underlying the results available to ensure full reproducibility?

Yes

Is the study design appropriate and is the work technically sound?

Yes

Are the conclusions drawn adequately supported by the results?

Yes

Are sufficient details of methods and analysis provided to allow replication by others?

Yes

Reviewer Expertise:

HR, Hotel Management, Tourism

I confirm that I have read this submission and believe that I have an appropriate level of expertise to confirm that it is of an acceptable scientific standard.
